# Aurora B inhibition induces hyper-polyploidy and loss of long-term proliferative potential in RB and p53 defective cells

**DOI:** 10.1038/s41419-024-07329-7

**Published:** 2025-01-08

**Authors:** Shivam Vora, Saptarshi Chatterjee, Ariel Andrew, Ramyashree Prasanna Kumar, Martina Proctor, Zhen Zeng, Rituparna Bhatt, Deborah Nazareth, Madushan Fernando, Mathew J. K. Jones, Yaowu He, John D. Hooper, Nigel A. J. McMillan, Jelena Urosevic, Jamal Saeh, Jon Travers, Daniela Cimini, Jing Chen, Brian Gabrielli

**Affiliations:** 1 Mater Research Institute, The University of Queensland, Brisbane, QLD Australia; 2 Department of Biological Sciences and Fralin Life Sciences Institute, Virginia Tech, Blacksburg, USA; 3 Frazer Institute, Faculty of Medicine, University of Queensland, Brisbane, QLD Australia; 4 School of Chemistry & Molecular Biosciences, University of Queensland, Brisbane, QLD Australia; 5 Menzies Health Institute Queensland and School of Medical Science, Griffith University, Gold Coast, Australia; 6 Research and Early Development, Oncology R&D, AstraZeneca, Cambridge, UK; 7 Research and Early Development, Oncology R&D, AstraZeneca, Waltham, USA; 8 Center for Soft Matter and Biological Physics, Virginia Tech, Blacksburg, USA

**Keywords:** Targeted therapies, Cytokinesis

## Abstract

Polyploidy is a common outcome of chemotherapies, but there is conflicting evidence as to whether polyploidy is an adverse, benign or even favourable outcome. We show Aurora B kinase inhibitors efficiently promote polyploidy in many cell types, resulting in the cell cycle exit in RB and p53 functional cells, but hyper-polyploidy in cells with loss of RB and p53 function. These hyper-polyploid cells (>8n DNA content) are viable but have lost long-term proliferative potential in vitro and fail to form tumours in vivo. Investigation of mitosis in these cells revealed high numbers of centrosomes that were capable of supporting functional mitotic spindle poles, but these failed to progress to anaphase/telophase structures even when AURKB inhibitor was removed after 2–3 days. However, when AURKB inhibitor was removed after 1 day and cells had failed a single cytokinesis to become tetraploid, they retained colony forming ability and long-term proliferative potential. Mathematical modelling of the potential for polyploid cells to produce viable daughter cells demonstrated that cells with >8n DNA and >4 functional spindle poles approach zero probability of a viable daughter, supporting our experimental observations. These findings demonstrate that tetraploidy is tolerated by tumour cells, but higher ploidy states are incompatible with long-term proliferative potential.

**Figure:**
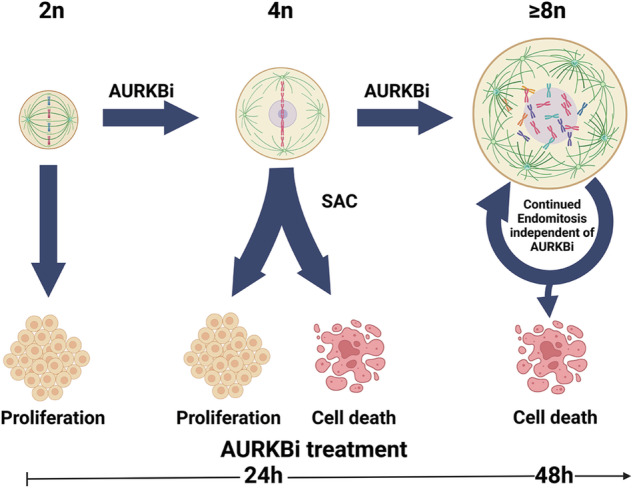
Model for AURKBi driven hyper-polyploid cells formation and fate. Aurora B inhibitor (AURKBi) treatment of RB+p53 defective cells efficiently promotes failed cell division. One failed cell division produces three possible outcomes, continued proliferation of the tetraploid daughter, cell death, or if AURKBi is continued, high polyploid states. Once cell have failed cell division >twice and have >8n DNA content they will continue to undergo rounds of endomitosis even in the absence of AURKBi to either become viable hyper-polyploid or die. The hyper-polyploid cells have no long-term proliferative potential.

Model for AURKBi driven hyper-polyploid cells formation and fate. Aurora B inhibitor (AURKBi) treatment of RB+p53 defective cells efficiently promotes failed cell division. One failed cell division produces three possible outcomes, continued proliferation of the tetraploid daughter, cell death, or if AURKBi is continued, high polyploid states. Once cell have failed cell division >twice and have >8n DNA content they will continue to undergo rounds of endomitosis even in the absence of AURKBi to either become viable hyper-polyploid or die. The hyper-polyploid cells have no long-term proliferative potential.

## Introduction

Polyploidy defines any cell with more than two sets of chromosomes, with the most common form of polyploidy being tetraploidy (4n). Multiple different mechanisms can promote polyploidy including endoreplication, where cells undergo rounds of replication without an intervening mitosis, endomitosis and cell fusion [1]. Polyploidy can be a physiologically controlled event [2], but is also a common feature of cancers and can be increased with chemotherapy [3]. Tetraploidy caused by genome duplication is often found in premalignant conditions [4, 5], and there is a direct, causative link between tetraploidy and tumorigenesis [6, 7]. In tumours, tetraploidy has been proposed to buffer changes in chromosome copy number, gene expression, mutations, and deletion [8]. Polyploid giant cancer cells (PGCCs) are a common feature of cultured cancer cell lines and observed in vivo, often as an outcome of chemotherapy [1, 9–12]. The contribution of PGCCs to cancer is unclear due to conflicting evidence pointing alternately to their tumorigenic or tumour suppressive potential. PGCCs have reported to be a hallmark of aggressive and treatment-resistant tumours [13–16], whereas other studies have reported that PGCCs are likely to undergo apoptosis [17] or enter therapy-induced senescence [18, 19], potentially a favourable therapeutic outcome [19].

Aurora kinase B (AURKB) inhibitors are known drivers of polyploidy [20]. AURKA and AURKB are functionally distinct regulators of progression through mitosis. AURKA is essential for centrosome maturation and regulates mitotic entry, whereas AURKB regulates exit from mitosis and controls correct partitioning of the replicated genome [20, 21]. AURKB inhibitors (AURKBi) have been investigated in a broad range of cancers, but none have yet been approved for clinical use [22]. Inhibition of AURKB disrupts chromosome alignment and segregation during mitosis, causes spindle assembly checkpoint override, and induces cytokinesis failure resulting in polyploidy [23–27]. Dual AURKA/B inhibitors were shown to cause a mitotic delay, the effect of AURKA inhibition, followed by slippage from mitosis and cytokinesis failure, the effect of AURKB inhibition [26–28]. The polyploidy resulting from failure of chromosome segregation and cytokinesis triggers a p53-dependent cell cycle arrest to prevent endoreplication [29]. However, the long-term outcomes of AURKBi treatment appear to be cell line dependent; cells may stop proliferating, become senescent, or undergo apoptosis [25, 28, 30–34]. We have recently reported that senescence triggered by AURKB inhibition is dependent on wild-type RB and p53, the p53 status determines the timing of senescence whereas intact RB is essential for senescence [35]. Loss of RB has also been reported to increase sensitivity to AURKB inhibitors [27, 32, 36].

Co-loss of RB and p53 function (RB+p53 defective) is a common feature of virally driven tumours such as HPV-driven cervical cancers, and found in >70% of small cell lung cancers [37]. Here we have investigated the outcomes of AURKBi treatment in RB+p53 defective tumour cell lines to determine whether the polyploid cells can undergo reductive cell divisions to produce viable daughter cells, and whether they have long-term proliferative potential as reported for PGCCs. We demonstrate that AURKBi efficiently promote the formation of hyper-polyploid (>8n DNA content) cells, and that these cells retain viability but not long-term proliferative potential. We also develop mathematical modelling supporting our experimental findings.

## Results

### Loss of RB1 function does not affect acute sensitivity to AURKBi in tumour cell lines

Studies have reported that RB defects are critical for sensitivity to AURK inhibitors [28, 32, 36]. To determine how universal this effect is, we analysed the Cancer Dependency Map (DepMap [38]) dataset for sensitivity to AURKA/Bi Alisertib and the AURKBi AZD2811 (Barasertib) from the Cancer Target Discovery and Development (CTD^2^ [39]) and Genomics of Drug Sensitivity (GDSC1/2 [40]) datasets that have sensitivity data for >1000 human cancer cell lines. 11% of cell lines contained RB1 mutations, the majority being destabilising mutations resulting in low RB protein levels (Supp. Figure S1A). RB1 mutant status did not influence sensitivity to either AZD2811 or Alisertib (Supp. Figure S1B, C). The lack of effect was not due to significant difference in the doubling time of the RB wild type and mutant cell lines (Supp. Figure S1D). RB mutations co-occur with p53 mutations in most cancers and cancer cell lines (TCGA Pan Cancer, CCLE; Supp. Figure S1E), but sensitivity to AURKBi was unaffected by combined low expression/mutation of RB+p53 mutation (Supp. Figure S1F). Over-expression of the pro-apoptotic BH3-only protein BID or anti-apoptotic BCL-2 family members have now been identified as major determinants of increased sensitivity to killing by AURKBi [41, 42].

### AURKBi promotes hyper-polyploidy in RB + p53 defective cells

**Fig. 1:**
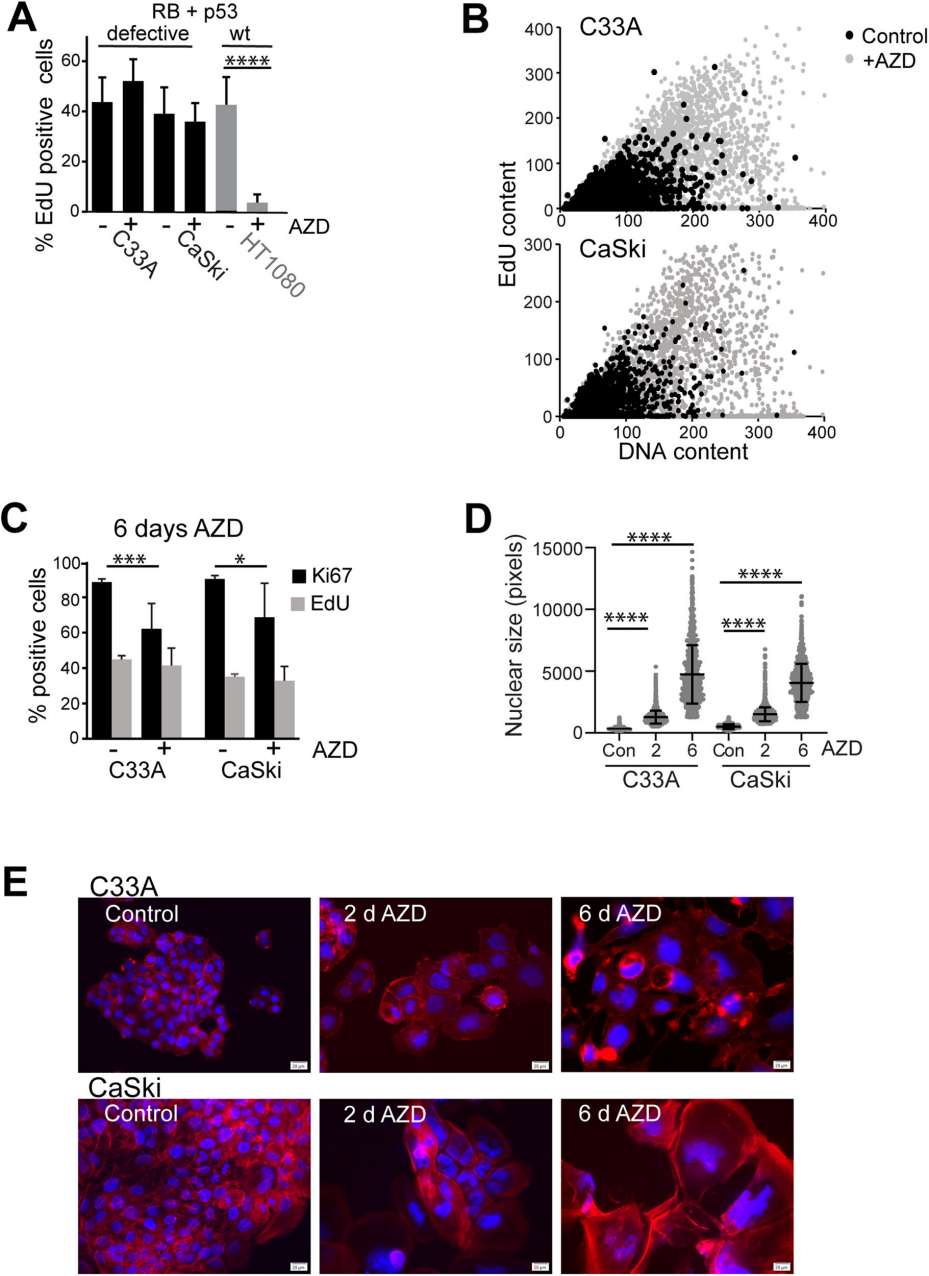
Loss of RB+p53 bypasses AZD2811-induced cell cycle arrest. **A** RB+p53 wild type HT1080 and RB+p53 defective C33A and CaSki cells were treated for 3 days with and without (control) 200 nM AZD2811 (AZD; AURKBi) then labelled for 2 h with EdU and stained for EdU incorporation. The percentage of EdU positive cells was assessed in triplicate samples each containing >1000 cells and analysed using an unpaired t-test. **B** C33A and CaSki cells were treated with 200 nM AZD2811 for 2 days then EdU labelled for 2 h, fixed, and stained for EdU and DNA, then analysed by high content imaging. Each dot represents data from a single cell. >4000 cells were analysed for each condition. **C** C33A and CaSki cells were treated with 200 nM AZD2811 for 6 days then EdU labelled for 2 h, fixed and stained for EdU and Ki67, then analysed by high content imaging. The data are the mean and standard deviation of triplicate wells each containing >1500 cells, Data compared using two-way ANOVA. **D** Nuclear size was obtained by quantifying nuclear area from high content imaging data. In each case >1500 cells were imaged; data compared using one-way ANOVA. ***p < 0.001, ****p < 0.0001. **E** C33A and CaSki cells were treated with 200 nM AZD2811 for the indicated time, fixed and stained for DNA (blue) and actin (red), then imaged. Scale bars, 20 μm.

We have previous shown that AURKB inhibition induces polyploidy in all cell lines investigated [35], but cell cycle exit, indicated by loss of EdU incorporation, was only observed in cells with functional RB and p53 (HT1080) and not in cells with loss of RB and p53 function (RB+p53 defective) due to mutation (C33A) or HPV E6/E7 expression (CaSki; Fig. 1A). High content imaging and flow cytometry revealed that RB+p53 defective C33A and CaSki cells became hyper-polyploid (>8n DNA content; Fig. 1B, Supp. Figure S2A), but continued to replicate their DNA, indicated by persistent EdU incorporation and Ki67-positivity (Fig. 1B, C). The decreased Ki67 staining and the larger error bars with treatment are likely to be a consequence of small number of very large cells imaged in each field. Nuclear size also increased with treatment (Fig. 1D), corresponding to increased cell size (Fig. 1E). Nuclear size increased modestly (20%) with treatment from day 2 to 6 in RB+p53 wild-type HCT116 cells, but increased almost 2-fold from 2 to 6 days treatment in HCT116 RB^−/−^p53^−/−^ cells (Supp. Figure S2B, C). Ki67 staining was reduced with treatment in the HCT116 wild-type cells, but only modestly reduced with 6-day treatment in HCT116 RB^−/−^p53^−/−^ cells (Supp. Figure S2C, D). These data support our previous findings that loss of RB+p53 function bypasses AURKBi-induced cell cycle arrest [35].

**Fig. 2:**
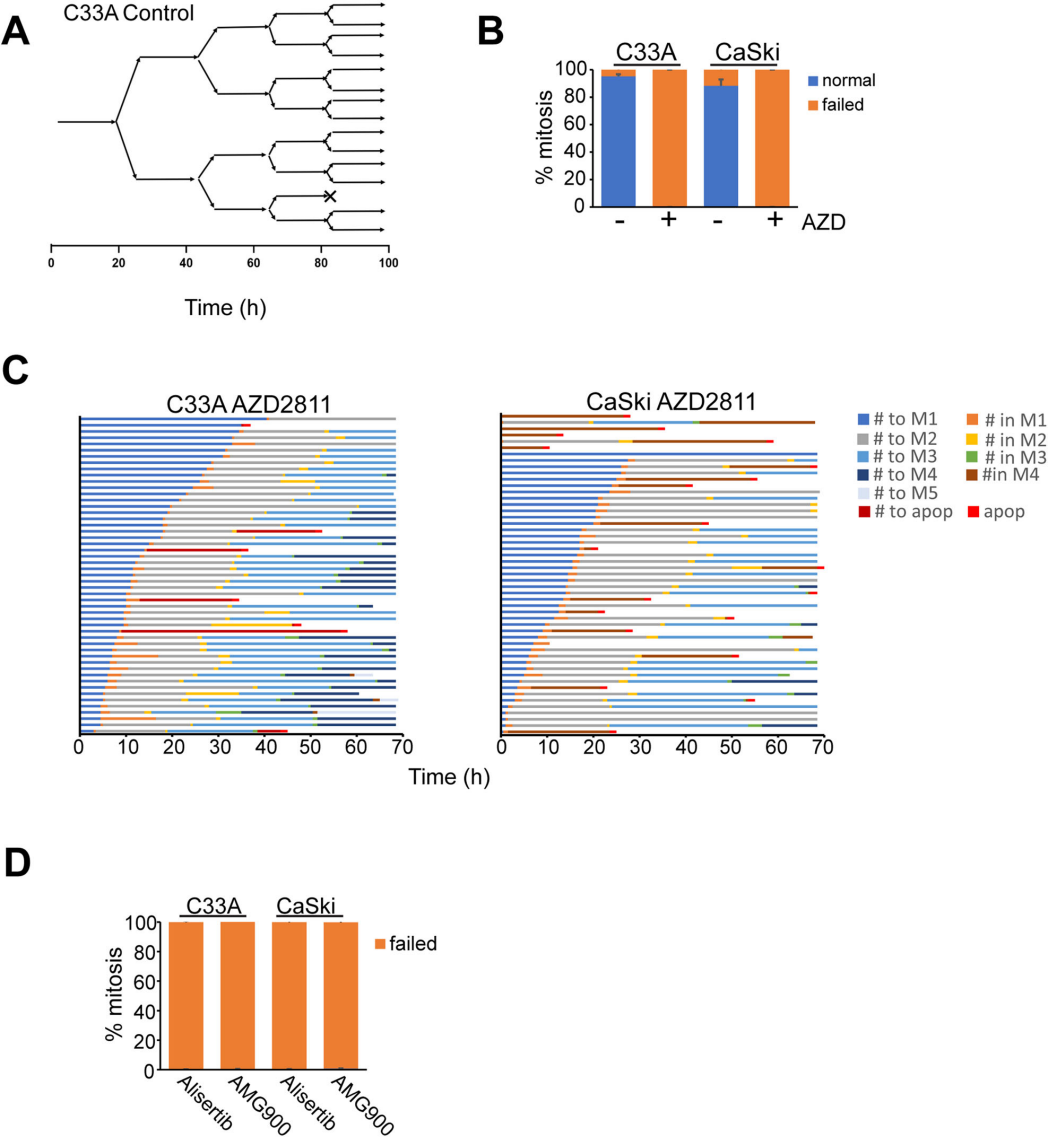
AURKBi induces persistent cell division failure. **A** Timeline of a single C33A cell followed for 3 days using time lapse microscopy. It shows that the majority of divisions resulted in two daughter cells. **B** The percentage of normal and failed cell divisions obtained from 3-day time lapse imaging of C33A and CaSki cells without and with 200 nM AZD2811 treatment. The data are from >50 cells for each condition. **C** Timelines for C33A and CaSki cells treated with AZD2811 as in (**B**) and followed for 3 days. **D** C33A and CaSki cells treated with either 1 μM alisertib or 300 nM AMG900 and followed for 3 days by time lapse microscopy. The percentage of failed cell divisions was determined for >50 cells per condition.

To assess the efficiency of AURKBi in promoting polyploidy, we performed time lapse imaging of C33A and CaSki cells treated with AZD2811 over 3 days. Over this time, control cells undergo 3–4 mitoses, with >90% producing two daughter cells via successful cytokinesis (Fig. 2A, B), whereas AZD2811-treated cells entered mitosis up to four times over the three-day imaging window but failed to successfully divide (Fig. 2B, C). The effects of AZD2811 were due to inhibition of AURKB as two other AURK inhibitors, alisertib and AMG900, which inhibit both AURKB and AURKA, produced similar rates of cell division failure over the same three-day period (Fig. 2D).

### Hyper-polyploid cells are incapable of producing proliferative subclones

**Fig. 3:**
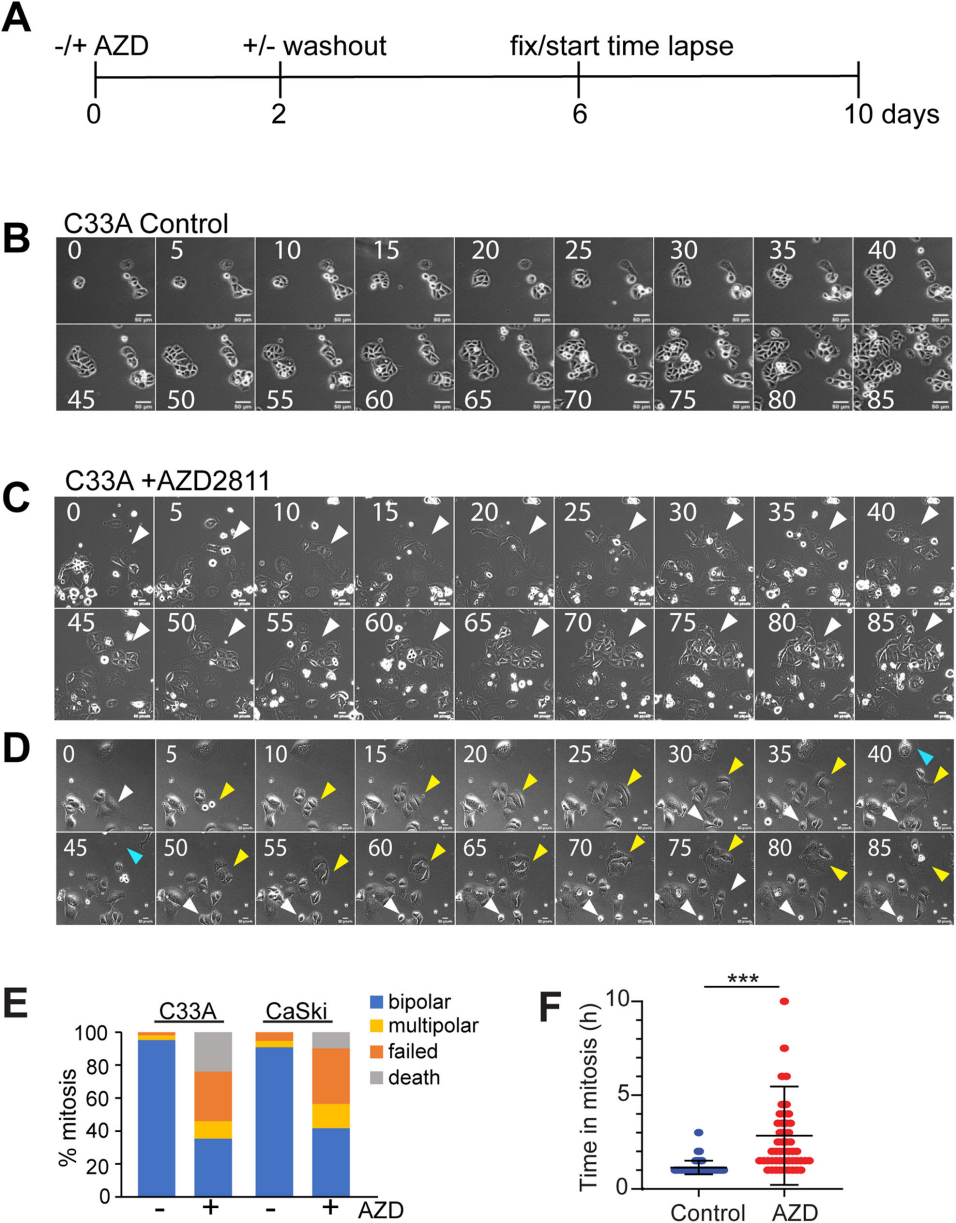
AZD2811 treated cells have no long-term proliferative potential. **A** Timeline of the experiment. Time lapse images of **B** control C33A cells, **C**, **D** C33A cells treated with 200 nM AZD2811. Cells were treated for 2 days then the drug was removed, and the cells were imaged from day 6 to 10. Images were collected every 30 min, and montage shows still images at 5 h intervals. Bars indicated 50 μm. **E**. Outcomes of cell division from the time lapse shown in (**A**–**D**) and a parallel experiment using CaSki cells. 100 cells were analysed for each condition. **F** Mitotic duration for C33A cells treated for 2 days with 200 nM AZD2811 then washed off and followed by time lapse microscopy from day 6 to 10. Statistical significance was assessed using a t-test; ***p < 0.001.

We next asked whether the hyper-polyploid cells induced by AURKB inhibition had the ability to undergo reductive mitosis to produce proliferative daughter cells as suggested for PGCCs [1, 9–12]. C33A and CaSki cells were treated with AZD2811 for 2–3 days, then drug washed out and incubated in drug-free media, and time lapse imaging was performed from days 6–10 after commencement of treatment (Fig. 3A). Control cells proliferated normally, whereas AZD2811-treated cells continued to progress through endomitotic cycles and fail cell division at high rates (~40%, Fig. 3B–E; Supp. Movies 1–3). A small fraction of large cells underwent multipolar division to produce multiple daughter cells, either three or more daughters of similar size, or one large and one or two much smaller daughter cells (Fig. 3C, arrowhead; Fig. 3D yellow arrowhead). The common outcome of this was death of all daughters (Fig. 3D, white arrowhead; Fig. 3E). The ~40% of bipolar cell divisions in AZD2811-treated cells (Fig. 3E) was contributed by a small fraction of large cells that undergo several rounds of cell division (e.g. Figure 3C, arrowhead). Many of the large multinucleated cells died during imaging (Fig. 3D, cyan arrowhead), accounting for 10–20% of the cells undergoing mitosis (Fig. 3E). Mitosis in these large polyploid cells was clearly perturbed as cells delayed in mitosis for extended periods (>3 times the average normal mitosis Fig. 3F), a phenomenon typically observed in cells that slip out of mitosis without proper chromosome segregation [43].

Immunofluorescence microscopy of the 6-day treated C33A cultures revealed that the very few colonies of smaller cells had an increased number of spindle poles (Figure S3A). The large polyploid mitotic cells formed asymmetric structures which were likely to be forerunners of the asymmetric cytokinesis observed in the time lapse sequences including the formation of small buds that contain little or no DNA (Figure S3B, C; Supp. Movie 2 yellow arrowhead).

**Fig. 4:**
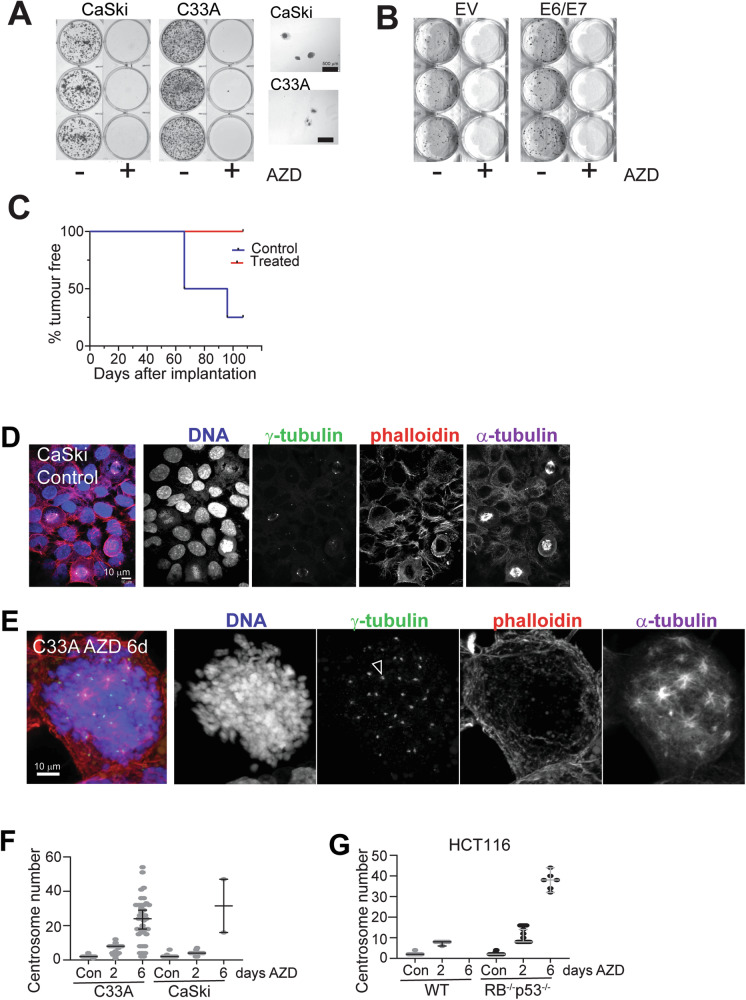
AZD2811 treated cells have no colony forming or tumorigenic potential. **A** Colony formation assays with the indicated cell lines in which cells were either untreated or treated for 3 days with AURKBi, then washed out, and plated to allow colony formation. **B** Similar experiment to (**A**) using immortalised human keratinocytes (EV) or EV cells expressing HPV E6/E7. **C** Nude mice were injected with either untreated or 5-day AZD2811 treated HCT116 RB^−/−^p53^−/−^. When the tumours were >100 mm^3^, the mice were culled, sacrificed, and examined upon autopsy. **D** Confocal microscopy of control C33A cells stained for DNA, γ-tubulin, α-tubulin to mark centrosomes and mitotic spindles and phalloidin for the actin cytoskeleton. **E** 6 day 200 nM AZD2811 treated mitotic cells. The bar in each is 10 μm. **F** C33A cell treated with 200 nM AZD2811 for the indicated times and stained as in (**D**, **E**) were visually inspected and centrosome number of the mitotic cells at each time point counted. **G** Parallel experiment to (**F**) but using either HCT116 wild type or RB^−/−^p53^−/−^ cells. The data in (**C**) and (**D**) show mean and 95% confidence intervals.

Despite hyper-polyploid cells continuing to undergo mitosis, the confluence of cultures rarely increased during imaging when compared to control cells, possibly due to a combination of failed mitotic divisions and cell death. This was confirmed by colony formation assays with CaSki and C33A cells that revealed no colonies formed when cells were treated for the first three days with AURKBi, although large individual cells were observed (Fig. 4A). Similarly, immortalised human keratinocytes expressing HPV E6/E7 which suppress RB and p53 function [44], failed to form colonies when treated with AURKBi (Fig. 4B).

To investigate whether the AURKBi-induced hyper-polyploid cells can generate viable daughter cells in vivo, parental HCT116 RB^−/−^p53^−/−^ cells, and hyper-polyploid cells derived from 5 days AZD2811 treatment were injected into nude mice for an in vivo tumour formation assay. Three million viable parental or AURKBi hyper-polyploid cells were injected in flanks of nude mice (the hyper-polyploid cells were injected into two sites due to their > 10-fold larger cell volume) and tumour growth monitored. Despite the long tumour growth delay, tumour growth from untreated cells was observed in 3 of 4 controls by 107 days, but no tumour formation resulted from injection of AURKBi-treated hyper-polyploid cells even after 107 days (Fig. 4C). Autopsy failed to reveal tumour growth at any sites within the mice. The lack of tumour growth of AURKBi-induced hyper-polyploid cells contrasts with the reported increased tumorigenicity of PGCCs [1, 15].

### AURKBi-induced hyper-polyploidy results in amplified centrosome numbers

To determine whether the generation of hyper-polyploid cells was due to a defect in centrosome duplication in response to prolonged AURKB inhibition, we immunostained centrosomes and microtubules. As expected, control cells displayed bipolar spindles with one centrosome at each spindle pole (Fig. 4D). Conversely, the large cells emerging as a result of a six-day AZD2811 treatment contained multiple centrosomes that formed individual spindle poles in mitosis spaced throughout the cell, although occasional clusters of two centrosomes in a single spindle pole were observed (Fig. 4E, Movie 4). Quantification of centrosome numbers showed that the majority of control cells had two centrosomes, whereas after 2 days of treatment, centrosome numbers increased to means of 4-11, depending on the cell line, possibly due to differences in proliferation rates (Fig. 4F, G). Interestingly, the few mitotic cells found in the HCT116 wild-type cells (RB and p53 wild type) after two days of treatment had a mean of 8 centrosomes, indicating these cells had undergone two rounds of failed cell division. After six days of treatment, C33A cells had an average of 32 centrosomes, whereas the few mitotic CaSki and HCT116 RB^−/−^p53^−/−^ cells that were found had an average of 23 and 38, respectively. No mitotic cells were found in the HCT116 wild-type cells at day six.

The time lapse and colony formation experiments had been performed on cells incubated with drug for only 2–3 days then drug removed, suggesting 2–3-day treatment was sufficient to promote continued failed cell divisions even in the absence of AURKBi. Immunofluorescence microscopy revealed that cells treated with AURKBi for 2 days then media replaced with fresh media without drug for another 4 days displayed similarly amplified centrosomes capable of forming multiple spindle poles in mitotic cells (Figure S4). This indicated that even in the absence of AURKBi, cells that had become hyper-polyploid continued to undergo endomitotic cycles.

### Tetraploid cells retain long-term proliferative potential

**Fig. 5:**
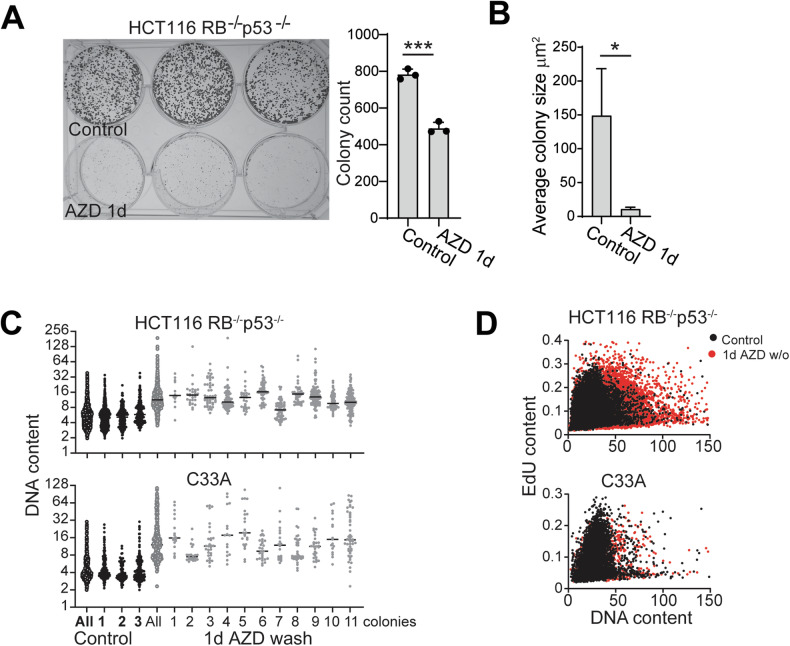
Tetraploid cells retain long-term proliferative potential. **A** Colony formation assay with HCT116 RB^−/−^p53^−/−^ control and 1 day AZD2811 treated then washed off cells. The number of colonies in each of the replicate wells was counted and shown as mean and SD. ***p > 0.001, unpaired t-test. **B** Size of the colonies from control and 1 day AZD-treated cells from (**A**). *p > 0.05, unpaired t-test. **C** DNA content of individual colonies of HCT116 RB^−/−^p53^−/−^ and C33A cells from parallel experiment to (**A**). **D** Cells grown from the combined colonies in a similar experiment to (**A**), were labelled with EdU, fixed and stained for EdU incorporation and DNA content.

Tetraploidy is a common outcome in response to physiological stress and is a widely acknowledged cancer-promoting mechanism [8]. We predicted that tetraploid cells were more likely to generate proliferative clones than cells with DNA content >8n. We treated RB+p53 defective cells with 200 nM AZD2811 for either 1 (1 failed cell division) or 3 days (>2 failed cell divisions), then the drug was removed, and colony formation assessed. One-day treatment significantly reduced colony formation, and the colonies were significantly reduced in size (Fig. 5A, B), whereas no colonies were found in the 3-day/wash-out plates (Fig. 4A, B). The DNA content of the individual colonies from 1 day-treated cells were on average tetraploid, although the ploidy of individual colonies varied considerably (Fig. 5C). When the cells were allowed to continue proliferating, HCT116 RB^−/−^p53^−/−^ cells retained proliferative tetraploid cells, whereas the proliferative C33A cells predominantly had original ploidy (Fig. 5D, Figure S5). This suggests that tetraploidy is overall well tolerated by cancer cells with loss of RB and p53 function.

### Mathematical model explains contrasting mitotic fate in tetraploid versus hyper-polyploid cells

**Fig. 6:**
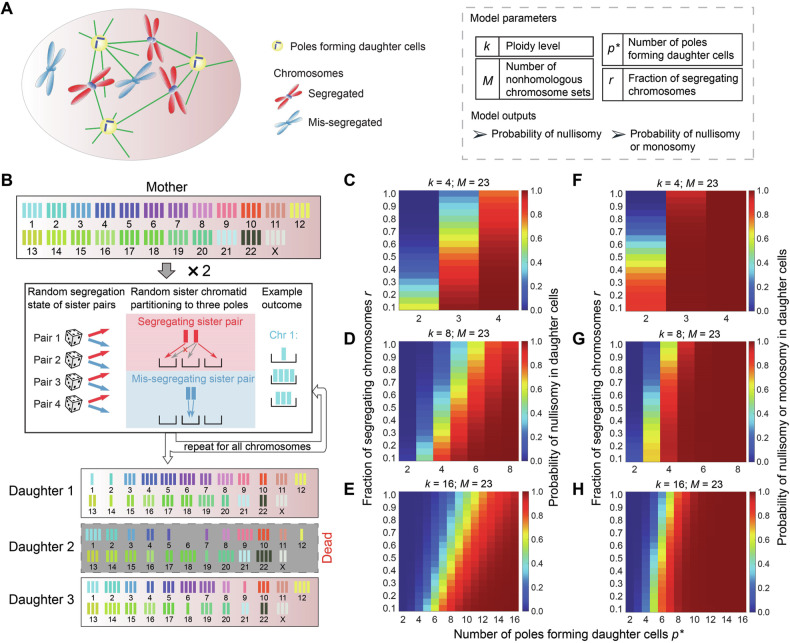
Mathematical modelling of polyploid mitosis outcomes. **A** Cartoon illustration of chromosome partitioning during multipolar divisions. The cell could contain a subset of non-segregating chromosomes. Model parameters and outputs are listed in the box. **B** A probabilistic model evaluates the karyotypic outcomes of multipolar divisions. Illustrated within a tripolar division of a tetraploid cell, chromosomes undergo random distribution among three poles. Each chromosome has a certain probability *r* to successfully segregate. Segregating chromosomes have their two sister chromatids partitioned to different poles. Non-segregating chromosomes have both sister chromatids partitioned to the same pole. In the example outcome, Daughter 2 is dead because chromosome 6 bears nullisomy. **C**–**H** The predicted probability of nullisomy (**C**–**E**) and that of nullisomy or monosomy (**F**–**H**) are sensitive to the fraction of segregating chromosomes *r*, effective number of daughter-forming poles p*, and ploidy level *k*. Results shown for various ploidy levels in human cancer cells (with 23 chromosomes per haploid set). Data for *k* = 32, 64 in Figure S6.

The experimental data has shown complete lack of proliferative potential of hyper-polyploid (≥8n) cells compared to a reduced proliferative potential of tetraploid (4n) cells. A reason behind these distinct outcomes could be the higher probability of failed mitosis due to large number of spindle poles formed in hyper-polyploid cells. The possibility of a single spindle pole being able to find and attach to a viable set of individual chromosomes and avoid fatal karyotypic deficiencies such as nullisomy or monosomy is expected to reduce rapidly with the increasing number of functioning spindle poles. However, increasing ploidy of the mother cell could attenuate the above deficiencies. Hence it is not immediately clear whether daughters of hyper-polyploid cells are likely to suffer fatal karyotypic deficiencies. To evaluate this likelihood, we built upon an existing mathematical model [45] and calculated the probability of unviable karyotypes arising from multipolar divisions across cells of different ploidy levels. Our model considers a *k*-ploid cell division with *p* spindle poles and M non-homologous sets of chromosomes (Fig. 6A, B).

We made the following assumptions while framing the current model:

1. All chromosomes are assumed to behave equivalently in partitioning during cell division and are independent of each other. Normally, sister chromatids are segregated by two randomly selected spindle poles.
2. Some chromosomes could be mis-segregated. In large hyper-polyploid cells particularly, steric hindrance will impede some chromosomes from attaching to spindle poles [46], enhancing the likelihood of chromosome mis-segregation. Considering these factors, the model is generalized to allow a subset of sister chromatids to be unsegregated and partitioned into the same daughter cells (Fig. 6A, B). Each individual chromosome is assumed to have probability *r* of normal segregation. (Note that in this model we do not consider mis-segregation events that affects the integrity of a chromatid, e.g., chromosome bridges that undergo breakage during segregation.)
3. Cytokinesis could be incomplete, especially with too many spindle poles. Some spindle poles may fail to separate into different daughter cells. We generalize the model such that with a total of *p* poles, only *βp* daughter cells form (0 ≤ *β* ≤ 1). We rename *βp* as p*, which is the effective number of poles forming daughter cells (Fig. 6A).

Utilizing the model, we evaluated how the probability of fatal karyotypes (nullisomy or nullisomy/monosomy) resulting from mitosis of a *k*-ploid human cell (*k* = 4, 8, 16 and *M* = 23) depends on the fraction of segregating chromosomes *r* and the number of functioning poles p* (Fig. 6A, B). Our results show that for a constant value of *r* in a *k*-ploid mitosis, the likelihood of nullisomy rises as the number of functioning spindle poles increases (Fig. 6C, E, Figure S6A, B; Eq. 8). Furthermore, decreasing value of *r* raises the likelihood of nullisomy (Fig. 6B–D, Figure S6C, D; Eq. 8). The model shows that for tetraploid cells, there is a significant reduction in the viability of daughter cells resulting from a tetrapolar division, whereas bi- and tripolar divisions produce daughter cells with more modest reduction in viability (Fig. 6C); this explains the experimental observations of reduced proliferative capacity in tetraploid cells (Fig. 5A). Monosomy may also be fatal and similar results (Fig. 6F–H; Eq. 22) as those for the probability of nullisomy were observed. The red, high-risk regions of mitotic fatality are larger for nullisomy/monosomy (Fig. 6F–H, Figure S6C, D) than nullisomy alone (Fig. 6C–E, Figure S6A, B), because the former case encompasses the latter.

Based on our model predictions, as well as the elevated likelihood of chromosome misattachments (due to steric hindrance) and lack of centrosome clustering (Fig. 4E) with increasing ploidy, hyper-polyploid cells are expected to have a higher probability than tetraploid cells to produce unviable daughters if they complete a multipolar division (Fig. 6I). The surviving hyper-polyploid cells completely failed to divide, slipped out of mitosis after a mitotic delay, and underwent repeated endomitoses (Fig. 3F). Taken together, the above factors could explain the loss of long-term proliferative potential of cells after 2–3 days AURKBi treatment.

## Discussion

Here we have demonstrated that AURKB inhibition in RB+p53 defective cancer cells, irrespective of their different genetic backgrounds results in the formation of hyper-polyploid cells that do not have long-term proliferative capacity either in vitro or in vivo. This contrasts with PGCCs which have been claimed to represent reservoirs of aggressive and treatment-resistant cancer cells [13–16]. AURKB inhibitors have been used in many clinical trials [47] and induce polyploidy in vivo [48] but no evidence they promote more aggressive disease has been reported. In most studies, PGCCs were generated by treatment with chemotherapeutic drugs resulting in a single failed mitosis [18, 49–53]. The PGCCs have been shown to undergo nuclear fragmentation and nuclear budding [18, 51], similar to events we observed with the AURKB inhibitor, and these were suggested to be mechanisms responsible for producing cell progeny with reduced ploidy. Specifically, it was suggested that the process of depolyploidization may involve segregation of the replicated genome via multipolar mitosis resulting in reductive mitosis producing multiple potentially non-identical daughter cells [18, 50, 54, 55]. However, our modelling work and previous studies on tetraploid cell populations [45] indicate that tetra-polar divisions can produce infrequent viable daughter cells with long-term proliferative potential, whereas higher ploidy multipolar spindles have approaching zero potential of producing viable daughter cells. Therefore, it is more likely that the viable proliferative PGCCs may represent tetraploid cells that can potentially undergo reductive division to produce daughter cells with genomic contents different from the diploid parental cells. Although multipolar division in tetraploid cells often results in cell cycle arrest or death of the progeny, cells with proliferative capacity can arise [45]. This can explain our observation that, although 1-day treatment with AZD2811 reduced cell proliferation and possibly proliferative capacity, viable cells with long-term proliferative potential where produced. This can also explain the causative role of tetraploidy in tumorigenesis [6, 7] and why tetraploid, but not tumours with higher ploidies, are common in cancer [3].

Our findings also provide evidence that viable daughter cells with reduced ploidy cannot be produced from mitotic hyper-polyploid cells. Indeed, we found that once ploidy reaches ≥8n, cells become incapable of normal cell division and instead undergo endomitotic cycles. Mitotic hyper-polyploid cells displayed a mitotic delay, suggesting spindle assembly checkpoint activation, but eventually slip out of mitosis. The spindle checkpoint activation is likely due to the inability of microtubules from the multiple spindle poles to reach and bind to chromosomes due to steric hindrance from the very large chromosome mass [46]. In addition, the mixing of the hyper-polyploid chromosomes and very large chromosome mass would make the possibility of the microtubules from an individual spindle pole being able to successfully bind a viable complement of chromosomes then undergo a successful cytokinesis exceedingly remote.

In summary, AURKBi treatment of RB+p53 defective tumour cells efficiently promotes hyper-polyploidy, treatment for at least 2 failed cell divisions is sufficient to drive hyper-polyploidy, and these hyper-polyploid cells cannot undergo reductive mitosis and do not produce daughter cells with long-term proliferative potential.

## Materials and methods

### Cell lines

The parental HCT116 (colorectal carcinoma) and CaSki and C33A cell lines were obtained from American Type Culture Collection (Manassas, VA, USA). HCT116 RB^−/−^p53^−/−^ produced from the original HCT116 p53^−/−^ line [56] using CRISPR CAS9 knockout (Vora et al., in press). HT1080 (fibrosarcoma) cell line was purchased from CellBank Australia (Westmead, NSW, Australia). The cell lines were cultured as described previously [32]. Human cervical keratinocytes (HCK) stably expressing TERT [57] parental and HPV E6/E7 expressing were provided by Professor Nigel McMillan from Griffith University (Gold Coast, QLD, Australia) and cultured in Keratinocyte Serum Free Medium (GIBCO) supplemented with Bovine Pituitary Extract (50 μg/ml) and Epidermal Growth Factor (5 ng/ml) as well as 0.035 mM of Pen/Strep and CaCl_2_. The cell cultures were maintained at 37 °C, in low oxygen (2% O_2_ and 5% CO_2_). All cultures were mycoplasma free.

### High content imaging

Cells were treated with AURKBi or DMSO control for the indicated time, labelled with 10 μM EdU for 2 h and then fixed and stained as previously [58]. Cells were then immunostained using an anti-human Ki-67 (Dako # M724001) antibody and nuclear DNA was stained using 4,6-diamidino-2-phenylindole (DAPI) stain. Plates were imaged using the IN Cell 6500HS imaging system (GE Healthcare); the images were analysed using Cell Profiler version 4.2.1 (Broad Institute of MIT and Harvard) and data processed using either GraphPad Prism 8 or R Studio as described previously [58].

### Immunofluorescence

Cells grown on glass coverslips, permeabilised and blocked as described [58]. Cells were immunostained with antibodies or γ-tubulin (Santa Cruz Biotechnology #sc-17787), α-tubulin (Abcam #ab18251) and incubated with phalloidin and DAPI to stain the actin filaments and DNA, respectively. Cells were imaged using an Olympus FV3000 confocal microscope.

### Time lapse microscopy

Cells were seeded in a 12-well plate in triplicates and treated with DMSO (control) and AURKi. Live cell imaging was performed using the Zeiss Axio Observer 7 imaging system in 5% CO_2_ and 37 °C and imaged every 20 min for 6–10 days. Movies were analysed as previously [27].

### Colony formation assay

Cells were seeded 1000 cells per well into a 6-well plate and treated with either DMSO or AURKBi for either 24 h or 72 h then replaced with fresh media and incubated until colonies were observed. Colonies were fixed with 4% PFA and stained with 0.05% Crystal violet. Colonies from control and 1 day treated cells were stained with Hoechst 33372 to record nuclear DNA content prior to crystal violet staining.

### Mouse tumour assay

Animal experiments were performed with approved ethics from The University of Queensland Animal Ethics Committee (2022/AE000024). Six-week-old Nude Mice (ARC) were injected with 3 × 10^6^ control or 5d AURKBi treated cells in Matrigel. Tumour-free survival was measured, and the mice were culled when tumours were palpable.

Methods for the mathematical modelling and DepMap analysis are provided in the Supplementary Material.

## Supplementary information

Supplementary Material

Video 1 C33A Control

Video 2 C33A+AZD2811

VIdeo 3 C33A+AZD2811

Video 4 Z stack of 60X C33A+AZD2811 mitotic cell.

## Data Availability

All data generated or analysed during this study are included in this published article [and its supplementary information files].
