# Supplementary material for: Aurora B inhibition induces hyper-polyploidy and loss of long-term proliferative potential in RB and p53 defective cells
Source: Cell Death Dis. 2025 Jan 8;16(1):7. doi: 10.1038/s41419-024-07329-7 (PMC11711630; doi:10.1038/s41419-024-07329-7)

Flow cytometry

Cells were harvested and fixed as described previously [37]. The FACS analysis was conducted using the Beckman Coulter CytoFLEX-S. DNA content (PI signal) was analysed ungated, using the PE filter. Data was analysed on Flowjo (version 7.6.4, Becton, Dickinson & Company).

Analysis of DepMap data

Analysis of both the GDSC2 [43]and CTD^2 [42] small molecule viability datasets in DepMap was performed using the DepMap portal Data Explorer (http://www.depmap.org). Alisertib (MLN8237) was present in both datasets (725 cell lines in CTD^2; 404 cell lines in GDSC2), Barasertib (AZD2811) was present in the CTD^2 (725 cell lines). The area under the inhibition curves (AUC) was used for each dataset, with lower AUC indicating sensitivity to the drug. Expression, mutation, copy number and antibody staining intensity for all the cell lines was derived from the Cancer Cell Line Encyclopedia (CCLE; [66]). The sensitivity to Barasertib and Alisertib were highly correlated in the CTD^2 datasets (Spearman r = 0.67), as was the sensitivity to Alisertib in the cell lines overlapping between the CTD^2 and GSCD1 set (Spearman r = 0.46).

Mathematical modelling

General methodology:

Because chromosomes are assumed independent of each other, the likelihood of a karyotypic event (nullisomy, monosomy, or nullisomy/monosomy) in a daughter cell can be estimated based on the probability of that event occurring to any particular homologous set of chromosomes, such as Chromosome 1 (denoted as Chr1), without losing generality (Figure **7B**).

Let $P(\text{event in Chr1 in the cell})$ denote the probability of a particular event (e.g., nullisomy, monosomy, or nullisomy/monosomy) for Chr 1. The probability of no event occurring on Chr 1 in a daughter cell is $1 - P(\text{event in Chr1 in the cell})$. Since each chromosome set behaves independently, this probability applies to each chromosome set. Therefore, the probability of no event occurring to any chromosome set in a daughter cell is $1 - \big(1 - P(\text{event in Chr1 in the cell})\big)^M$ where M is the total number of non–homologous sets ($M = 23$ in this study). Hence, the probability of the concerned event occurring inside the cell will read as follows:

$$P(\text{event in the cell}) = 1 - \big(1 - P(\text{event in Chr1 in the cell})\big)^M. \quad (1)$$

Our goal is therefore to first estimate the probability of each event of interest for Chr1 and use that to determine the corresponding probability in the cell.

Probability of nullisomy

We define nullisomy as the situation where a particular pole/daughter cell receives no chromatids for a set of homologous chromosomes.

First of all, given the probability r of normal segregation, the number of segregating chromosomes out of k homologous chromosomes (e.g., Chr1) follows a binomial distribution and the probability of having q segregating chromosomes reads as follows:

$$P(q \text{ segregating chromosomes out of } k) = \binom{k}{q} r^q (1-r)^{k-q}. \quad (2)$$

Now, the total number of equally probable ways to segregate q pairs of sister chromatids and distribute the remaining $k - q$ unsegregated chromosomes to $p*$ effective poles reads as:

$$N_{\text{tot}} = N_{q\times 2 \to p*} \times N_{(k-q)\times 1 \to p*} = \binom{p^*}{2}^q (p^*)^{k-q}. \quad (3)$$

If a particular pole receives no chromatids (i.e., nullisomy), then the total number of equally probable ways to segregate q pairs of sister chromatids and distribute the remaining $k - q$ unsegregated chromosomes among the remaining ($p* - 1$) effective poles can be expressed as:

$$N_0 = N_{q\times 2 \to (p*-1)} \times N_{(k-q)\times 1 \to (p*-1)} = \binom{p^* - 1}{2}^q (p^* - 1)^{k-q}. \quad (4)$$

Hence, the probability of nullisomy in Chr1 based on q segregating chromosomes out of a total of k is:

$$P(\text{nullisomy in Chr1}|q \text{ segregating chromosomes out of } k)$$

$$= \frac{N_0}{N_{\text{tot}}} = \left(\frac{p^* - 2}{p^*}\right)^q \left(\frac{p^* - 1}{p^*}\right)^{k-q}. \tag{5}$$

In this scenario, the probability of nullisomy in Chr1 becomes,

$$P(\text{nullisomy in Chr1 in the cell})$$

$$= \sum_{q=0}^{k} \{P(q \text{ segregating chromosomes out of } k)$$

$$\times P(\text{nullisomy in Chr1}|q \text{ segregating chromosomes out of } k)\}$$

$$= \sum_{q=0}^{k} \binom{k}{q} r^q (1-r)^{k-q} \left(\frac{p^* - 2}{p^*}\right)^q \left(\frac{p^* - 1}{p^*}\right)^{k-q}. \tag{6}$$

Now let us rename $\left(\frac{p^* - 2}{p^*}\right)$ as γ and $\left(\frac{p^* - 1}{p^*}\right)$ as η. Subsequently, upon algebraic simplification and using binomial theorem, Equation (6) becomes,

$$P(\text{nullisomy in Chr1 in the cell}) = \sum_{q=0}^{k} \binom{k}{q} (\gamma r)^q \big(\eta(1-r)\big)^{k-q} = \big(\gamma r + \eta(1-r)\big)^k. \tag{7}$$

Plugging Equation (7) into Equation (1) and plugging back the definitions of γ and η give the probability of nullisomy in a cell:

$$P(\text{nullisomy in the cell}) = 1 - \left(1 - \left(\frac{p^* - 2}{p^*} r + \frac{p^* - 1}{p^*}(1-r)\right)^k\right)^M. \tag{8}$$

Probability of monosomy

We define monosomy as the situation where only a single chromatid is received by any pole for a set of homologous chromosomes. We again consider the scenario where q chromosomes out of a total of k homologs undergo segregation, leaving the remaining $k - q$ chromosomes unsegregated. The total number of equivalent ways to achieve monosomy in a particular pole is the product of the number of ways to choose one chromosome pair out of q pairs to partition one chromatid onto the pole, the number of ways to choose 1 pole out of the remaining $p^* - 1$ poles for the other chromatid of the monosomic pair, the number of ways to segregate the remaining $q - 1$ chromosome pairs onto the remaining $p^* - 1$ poles, the number of ways to

distribute the remaining $k-q$ unsegregated chromosomes to the remaining p^*-1 poles. That is,

$$N_1 = q \times (p^*-1) \times N_{(q-1)\times 2 \to (p*-1)} \times N_{(k-q)\times 1 \to (p*-1)}$$
$$= q(p^*-1)\binom{p^*-1}{2}^{q-1}(p^*-1)^{k-q}. \quad (9)$$

Hence, the probability of monosomy in Chr1 based on q segregating chromosomes out of a total of k is:

$$P(\text{monosomy in Chr1}|q \text{ segregating chromosomes out of } k)$$
$$= \frac{N_1}{N_{\text{tot}}} = \frac{2q}{p^*-2}\left(\frac{p^*-2}{p^*}\right)^q\left(\frac{p^*-1}{p^*}\right)^{k-q}. \quad (10)$$

Therefore, the probability of monosomy in Chr1 becomes,

$$P(\text{monosomy in Chr1 in the cell})$$
$$= \sum_{q=0}^{k}\{P(q \text{ segregating chromosomes out of } k)$$
$$\times P(\text{monosomy in Chr1}|q \text{ segregating chromosomes out of } k)\}$$
$$= \sum_{q=0}^{k}\binom{k}{q} r^q(1-r)^{k-q}\frac{2q}{p^*-2}\left(\frac{p^*-2}{p^*}\right)^q\left(\frac{p^*-1}{p^*}\right)^{k-q}. \quad (11)$$

Upon algebraic simplification, Equation (11) becomes,

$$P(\text{monosomy in Chr1 in the cell}) = \frac{2}{p^*-2}\sum_{q=0}^{k} q\binom{k}{q}\left(\frac{p^*-2}{p^*}r\right)^q\left(\frac{p^*-1}{p^*}(1-r)\right)^{k-q}. \quad (12)$$

Now for the ease of handling the algebraic factors, let us rename,

$$a = \frac{p^*-2}{p^*}r. \quad (13)$$

and

$$b = \frac{p^*-1}{p^*}(1-r). \quad (14)$$

With the definitions of a and b, Equation (12) becomes,

$$P(\text{monosomy in Chr1 in the cell}) = \frac{2}{p^* - 2}\sum_{q=0}^{k} q\binom{k}{q} a^q b^{k-q}. \quad (15)$$

Now, using binomial theorem we can write,

$$(a + b)^k = \sum_{q=0}^{k} \binom{k}{q} a^q b^{k-q}. \quad (16)$$

Differentiating Equation (16) with respect to a and subsequently multiplying by a, we obtain,

$$ak(a + b)^{k-1} = \sum_{q=0}^{k} q\binom{k}{q} a^q b^{k-q}. \quad (17)$$

Therefore, from Equation (15) and Equation (17) we obtain,

$$P(\text{monosomy in Chr1 in the cell}) = \frac{2}{p^* - 2} ak(a + b)^{k-1}. \quad (18)$$

Plugging the definitions of a and b back into Equation (18) yields,

$$P(\text{monosomy in Chr1 in the cell}) = \frac{2kr}{p^*}\left(\frac{p^* - 2}{p^*} r + \frac{p^* - 1}{p^*}(1 - r)\right)^{k-1}. \quad (19)$$

Plugging Equation (19) into Equation (1) gives the probability of monosomy within a cell:

$$P(\text{monosomy in the cell}) = 1 - \left[1 - \frac{2kr}{p^*}\left(\frac{p^* - 2}{p^*} r + \frac{p^* - 1}{p^*}(1 - r)\right)^{k-1}\right]^M. \quad (20)$$

Probability of nullisomy and/or monosomy

From the physiological perspective, monosomy alone is not a good metric of unviability. The more relevant metric is the probability of monosomy and/or nullisomy. In other words, our criterion for an unviable cell can be either having at least one nullisomy or having at least one nullisomy/monosomy. The latter can be easily adapted from the results for nullisomy and monosomy. As nullisomy and monosomy are exclusive outcomes for a given chromosome, such as Chr1, for a given pole, the probability of either nullisomy or monosomy in both the designated pole and its corresponding daughter cell for Chr1 can be expressed as:

$$
\begin{aligned}
&P(\text{nullisomy or monosomy in Chr1 in the cell}) \\
&= P(\text{nullisomy in Chr1 in the cell}) + P(\text{monosomy in Chr1 in the cell}) \\
&= \left(\frac{p^* - 2}{p^*} r + \frac{p^* - 1}{p^*}(1 - r)\right)^k + \frac{2kr}{p^*}\left(\frac{p^* - 2}{p^*} r + \frac{p^* - 1}{p^*}(1 - r)\right)^{k-1}. \qquad (21)
\end{aligned}
$$

By substituting Equation (21) into Equation (1), we obtain the probability of having nullisomy or monosomy within a cell:

$$
\begin{aligned}
&P(\text{nullisomy or monosomy in the cell}) \\
&= 1 - \left[1 - \left(\frac{p^* - 2}{p^*} r + \frac{p^* - 1}{p^*}(1 - r)\right)^k - \frac{2kr}{p^*}\left(\frac{p^* - 2}{p^*} r + \frac{p^* - 1}{p^*}(1 - r)\right)^{k-1}\right]^M. \quad (22)
\end{aligned}
$$

Substituting $M = 23$, $k = 4$, 8 and 16 into Equation (8) and Equation (22) while varying r and p^* ($2 \leq p^* \leq k$) leads to the phase diagrams depicted in Figure **7C**–**7H**. The colormaps in the plots presented in Figure **7C**–**7I** and Figure **S5** were rendered using imagesc function in MATLAB 2023a (The MathWorks, Natick, MA).

Supplementary Figure S1. A: Expression data for RB1 showing RNAseq and reverse phase protein array analysis of the cell lines analysed in the CTD^2 screen. The orange and red dots are mutant RB1. **B.** The DepMap sensitivity data for AZD2811 (AZD) and Alisertib (Alis) from the CTD^2 drug screen was analysed on the basis of presence or absence of RB mutation. The AUC values (area under the dose response curve) were used to determine sensitivity, the higher values indicate less sensitivity. The box and whiskers plot shows the AUC for each drug for all 729 cell lines analysed. **C.** The DepMap sensitivity data for Alisertib from the GDSC1 drug screen was analysed on the basis of presence or absence of RB mutation. The AUC values (area under the dose response curve) were used to determine sensitivity, the higher values indicate less sensitivity. The box and whiskers plot shows the AUC for each drug for all 725 cell lines analysed. The AUC values differ from the CTD^2 screen due the different method used to calculate the relative AUC. Statistical significance was determined by t-test. * $p<0.05$. **D.** Doubling time vs. sensitivity to AURKBi for cell lines in the CTD^2 screen. The line shows the mean of the doubling times. **E.** Mutation and depletion analysis of RB1 and TP53 from TGCA and CCLE databases using cBioPortal. **F.** The DepMap sensitivity data for AZD2811 (AZD) from the CTD^2 drug screen was analysed on the basis of RB expression (low expression is <2 log2 (TPM+1)) or mutation with or without p53 mutation. The box and whiskers plot shows the AUC for each drug for all 729 cell lines analysed.

Supplementary Figure S2. A: C33A and CaSki cells were treated with 200 nM AZD2811 for 3 days, fixed and stained for DNA, then analysed by flow cytometry. Control cells are shown as the red histogram, and replicate AZD2811 treated cultures are shown as blue and orange histograms. **B:** WT and $RB^{-/-}p53^{-/-}$ HCT116 cells were treated with 200 nM AZD2811 for the indicated time, fixed and stained for DNA then imaged. Scale bar, 50 μm. **C,D:** WT and $RB^{-/-}p53^{-/-}$ HCT116 cells were treated as in A, fixed and stained for DNA and Ki67 then analysed using high content imaging. The nuclear size and percentage Ki67 positive cells are shown. In each case >4,000 cells were imaged. Ki67 data represents triplicate wells. One-way ANOVA, ** $p<0.01$, *** $p<0.001$, **** $p<0.0001$.

Supplementary Figure S3. C33A cells treated with 200 nM AZD2811 for 6 days, fixed and stained as in Figure 4. **A:** Small colony of smaller cells with tripolar mitosis (arrowheads). **B:** Large hyper-polyploid mitotic cell with appearance of actin contractile ring (arrowhead). **C:** Large hyper-polyploid mitotic cell with an anucleate bud.

Supplementary Figure S4. CaSki and C33A cells were treated with 200 nM AZD2811 for 2 days, then the drug was washed out with fresh media and cells were allowed to grow for 4 additional days (2d AZD+4d) then fixed and immunostained for the indicated markers.

Supplementary Figure S5. Ki67 staining intensity in HCT116 $RB^{-/-}p53^{-/-}$ or C33A line, either untreated or treated for 1 day with 200 nM AZD2811 then washed out and allowed to grow in fresh media as in Figure 6C.

Supplementary Figure S6. The predicted probability of nullisomy and that of nullisomy or monosomy are sensitive to the fraction of segregating chromosomes r, effective number of functioning poles p^*, and ploidy level k. Results shown for various ploidy levels in human cancer cells (with 23 chromosomes per haploid set). Data for $k = 32, 64$ for the same model from Figure 7.

Supplementary Movie 1

Control, untreated C33A cells were followed for 4 days. Time lapse images were collected every 30 min. Movie corresponds to Figure **3B**.

Supplementary Movie 2, 3

C33A cells treated with 200 nM AZD2811 for 3 days then the drug was washed out and fresh media replaced. Cells were imaged from 6 days after the start of treatment to 10 days. Time lapse images were collected every 30 min. **Movie 2** corresponds to **Figure 3C**. **Movie 3** corresponds to **Figure 3D**.

Supplementary Movie 4

Z stack of confocal images corresponding to the maximum intensity project in **Figure 5B**.

Vora et al Supplementary Figure S1

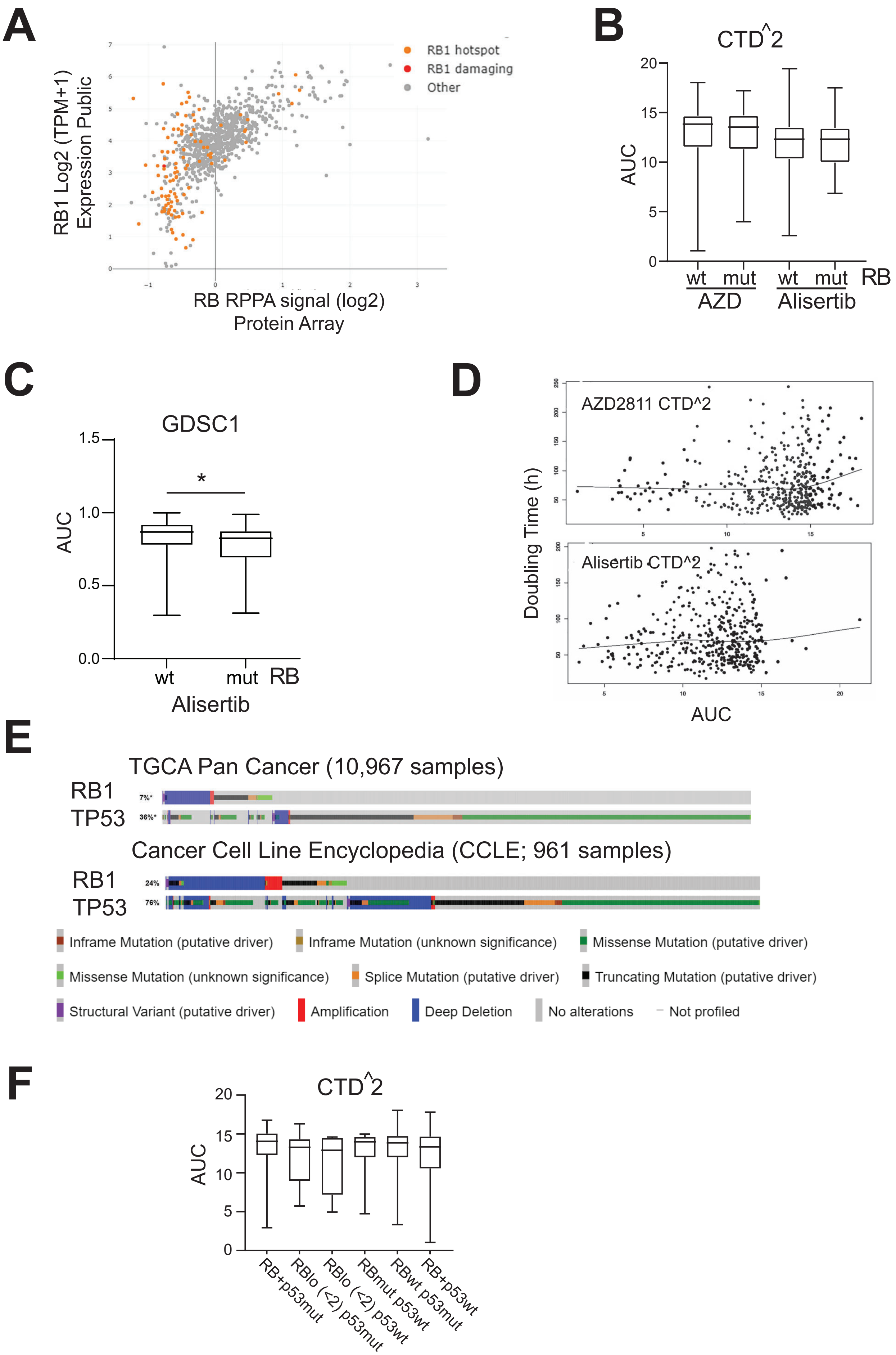

Vora et al Supp Figure S2

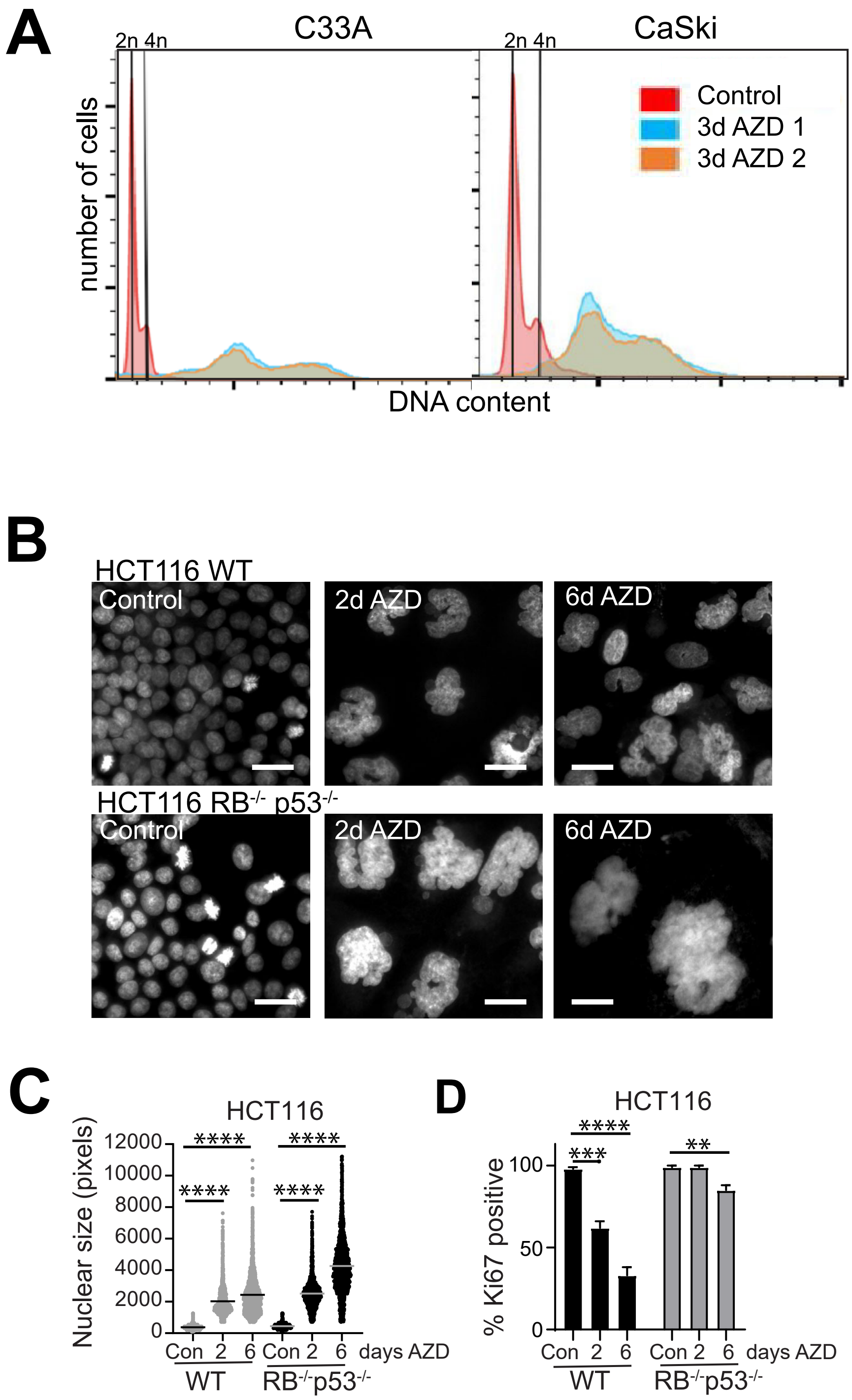

Vora et al Supplementary Figure S3

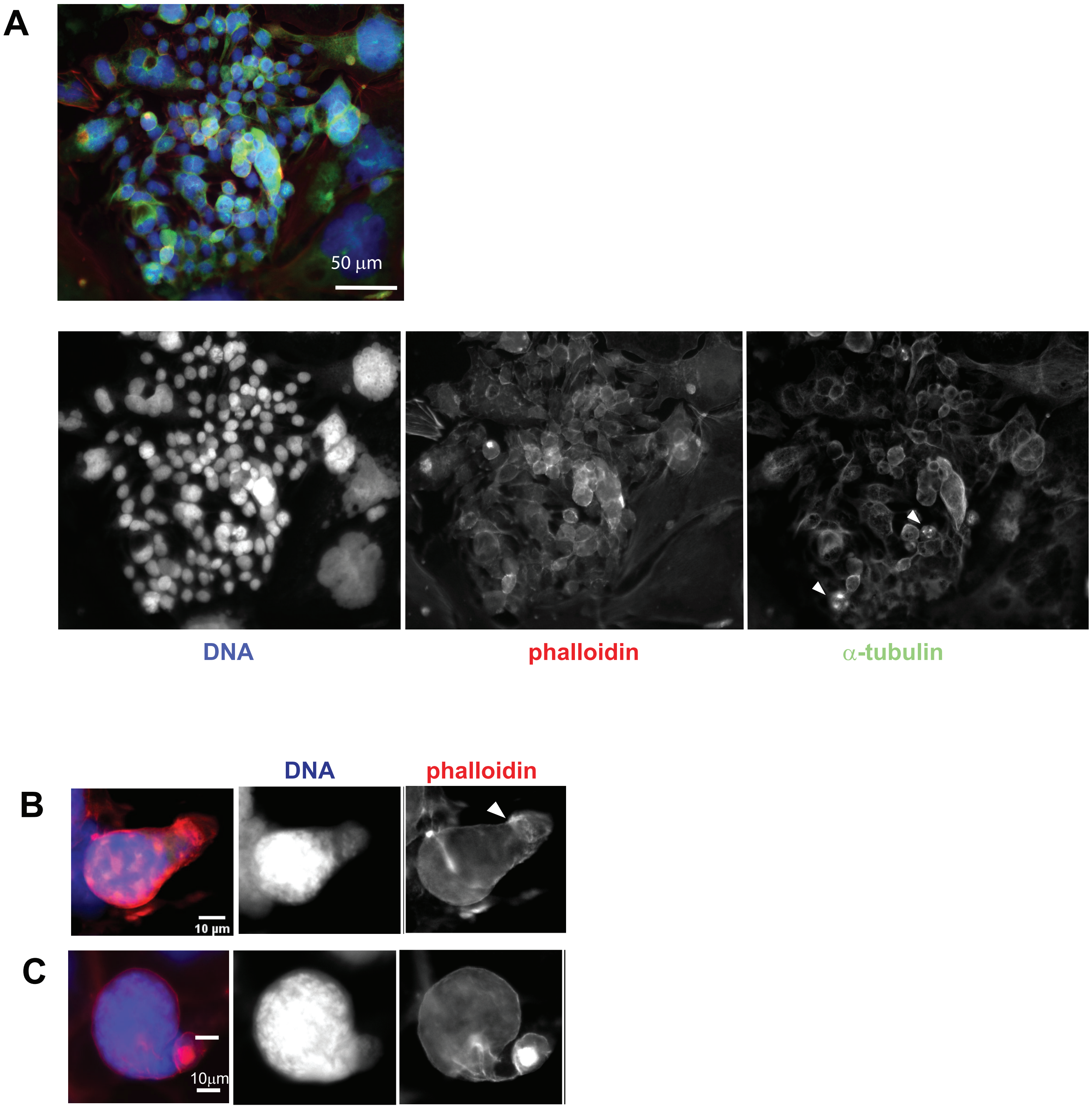

Vora et al Supplementary Figure S4

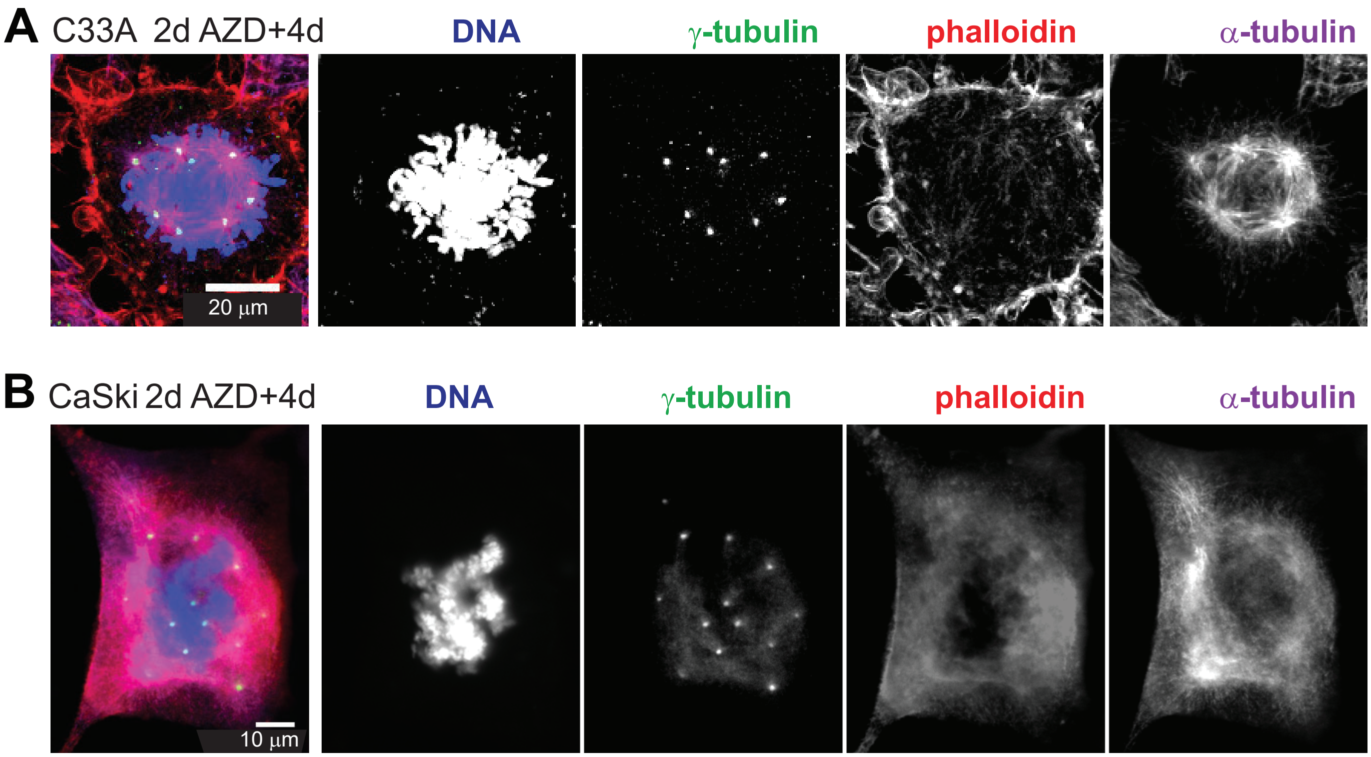

Vora et al Supplementary Figure S5

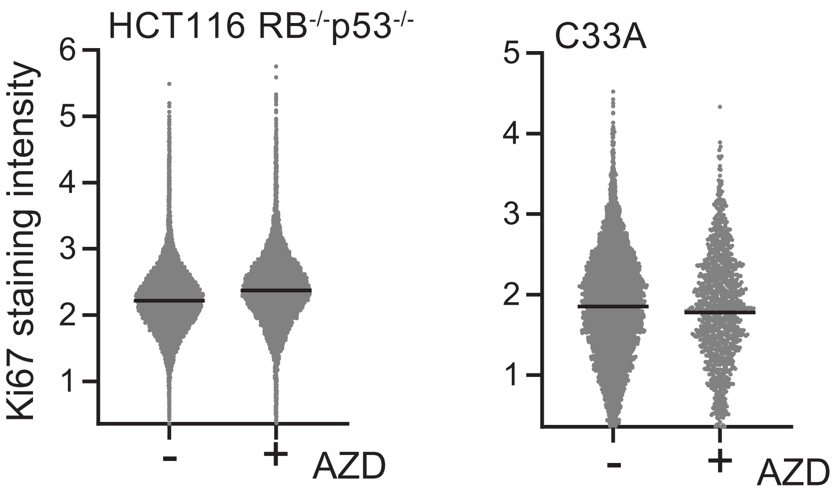

Vora et al Supp Figure S6

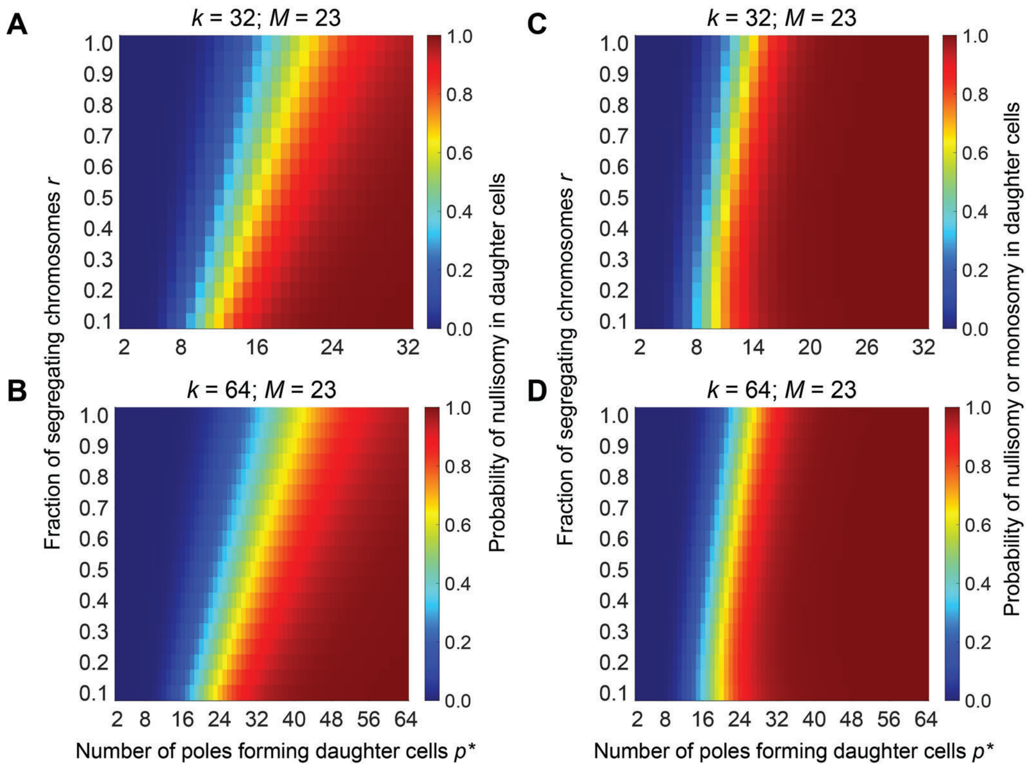

Supplement: Supplementary file 1 — Supplementary Material [file 41419_2024_7329_MOESM1_ESM.pdf]
